# Effect of Hepatic Pathology on Liver Regeneration: The Main Metabolic Mechanisms Causing Impaired Hepatic Regeneration

**DOI:** 10.3390/ijms24119112

**Published:** 2023-05-23

**Authors:** Svetlana Rodimova, Artem Mozherov, Vadim Elagin, Maria Karabut, Ilya Shchechkin, Dmitry Kozlov, Dmitry Krylov, Alena Gavrina, Nikolai Bobrov, Vladimir Zagainov, Elena Zagaynova, Daria Kuznetsova

**Affiliations:** 1 Institute of Experimental Oncology and Biomedical Technologies, Privolzhsky Research Medical University, 10/1 Minin and Pozharsky Sq., 603000 Nizhny Novgorod, Russiamaria.karabut@gmail.com (M.K.);; 2 Laboratory of Molecular Genetic Research, Institute of Clinical Medicine, N.I. Lobachevsky Nizhny Novgorod National Research State University, 23 Gagarina Ave., 603022 Nizhny Novgorod, Russia; 3 The Volga District Medical Centre of Federal Medical and Biological Agency, 14 Ilinskaya St., 603000 Nizhny Novgorod, Russia; 4 Nizhny Novgorod Regional Clinical Oncologic Dispensary, Delovaya St., 11/1, 603126 Nizhny Novgorod, Russia

**Keywords:** liver regeneration, liver pathology, metabolic mechanisms, FLIM

## Abstract

Liver regeneration has been studied for many decades, and the mechanisms underlying regeneration of normal liver following resection are well described. However, no less relevant is the study of mechanisms that disrupt the process of liver regeneration. First of all, a violation of liver regeneration can occur in the presence of concomitant hepatic pathology, which is a key factor reducing the liver’s regenerative potential. Understanding these mechanisms could enable the rational targeting of specific therapies to either reduce the factors inhibiting regeneration or to directly stimulate liver regeneration. This review describes the known mechanisms of normal liver regeneration and factors that reduce its regenerative potential, primarily at the level of hepatocyte metabolism, in the presence of concomitant hepatic pathology. We also briefly discuss promising strategies for stimulating liver regeneration and those concerning methods for assessing the regenerative potential of the liver, especially intraoperatively.

## 1. Mechanisms of Liver Regeneration

In a clinical setting, liver regeneration is associated with conditions that have led to severe loss of hepatocytes. Chronic loss of hepatocytes is seen in viral diseases, chronic damage (e.g., by alcohol, metabolic diseases, non-alcoholic steatohepatitis (NASH)) and ischemia-reperfusion injury, following liver transplantation, accompanied by compensatory proliferation of the surviving hepatocytes [1]. Acute loss of hepatocytes is often caused by acute toxic damage (e.g., by acetaminophen), trauma, or by acute hepatitis. In any case, compensatory proliferation of hepatocytes occurs in parallel with inflammatory processes that remove damaged hepatocytes and provide repair process [1,2].

Most of the known data on the mechanisms of liver regeneration have been obtained using the classical model of partial hepatectomy (PH), which allows standardization of the experimental conditions, since it is possible to accurately set the volume of tissue to be removed. This model is characterized by a high rate of liver recovery, is well tolerated by laboratory animals (traditionally rodents—rats and mice) and gives reliable results [3]. The most commonly used is 70% (or 2/3) PH involving removal of the left anterior and medial lobes of the liver. The 70% PH technique was first described by Higgins and Anderson in 1931 [4]. Normally, after 70% PH, up to 93% of the original liver volume is restored by 7–10 days for rats and 10–14 days for humans. Full regeneration of the starting volume of the liver is generally achieved by 20 days in rats and by 8 weeks in humans [5,6]. Less common is the 30% (or 1/3) PH model. A number of authors have shown that the main difference between these two models is the different mechanism of liver tissue repair. More specifically, when 2/3 of the liver is removed, hypertrophy of the remnant liver occurs rapidly, followed by hyperplasia, with entry of almost all hepatocytes into the S phase. In this context, binuclear hepatocytes undergo an unconventional cell division to cause two mononuclear daughter cells. In contrast, following 1/3 partial hepatectomy, the liver recovers its original mass mostly through hepatocyte hypertrophy alone, with few cell divisions and no modification of their ploidy [6]. In particular, Miyaoka et al. have highlighted the simultaneous presence of both hypertrophy and hyperplasia, with the predominance of hypertrophy. The authors conducted a comprehensive analysis of genetic fate mapping of liver cells and demonstrated that hepatocytes do enter the S phase, but it is not always followed by a normal M phase, so this ultimately results in an increase in the size of the hepatocytes [7]. Despite this, most authors confirm the proliferation of hepatocytes following PH [2,8,9]. Activation of various regeneration mechanisms is rather conditional, and in both models, activation of liver remnant cells from the G0 resting state to the replication phase occurs. At the end of the regenerative process, the non-resected lobes become larger and, in aggregate, restore the hepatic mass that was present prior to PH. The regenerated liver has a different shape; it now has fewer lobes (two or three, based on anatomic conventions), and the resected lobes are not restored [2].

Current knowledge indicates that the high regenerative potential of liver is ensured by the ability of hepatocytes to proliferate. Hepatocytes are highly differentiated, often polyploid cells that perform the numerous biochemical functions of the liver: metabolism of proteins, carbohydrates and fats, detoxification of xenobiotics and metabolic intermediates, synthesis of serum proteins, bile secretion and cholesterol excretion. A unique feature of hepatocytes is in their ability to perform synthetic functions while maintaining the ability to proliferate. Experimental models of repopulation and of transplantation of liver cells have shown that resting hepatocytes of a healthy liver are capable of almost unlimited proliferation [10].

An important role in the process of regeneration is played by the polyploidy of a proportion of the hepatocytes, including causing the rapid activation of hepatocyte proliferation in response to damage. Unlike most tissues that have cells with diploid genomes, the liver in many species of mammals, including humans, contains a mixed population of diploid and polyploid cells that differ in the number of chromosome sets. In the postnatal period, the process of mitotic polyploidization occurs in the liver, during which some hepatocytes become polyploid [11]. These daughter cells can then produce a pair of mononucleate tetraploid daughter cells with a round of cell cycling that includes mitosis, followed by successful cytokinesis [12].

The most pronounced polyploidy of liver cells is seen in rodents. According to current knowledge [13], the multiplication of the hepatocyte genome is an adaptation to accumulating unrepairable DNA damage. However, in humans, polyploidy in the liver is much less pronounced, the proportion of polyploid cells being 30–40% [14]. Postnatal growth of the human liver is carried out mainly by mitotic divisions of mononucleate, diploid hepatocytes, the number of which, in people up to 50 years of age, comprises almost 90% of the total. By the age of 80–90, about 15% binucleate tetraploid cells (2n × 2) and 10% mononucleate tetraploid cells (4n) have accumulated in the liver. The most polyploid hepatocytes in the human liver are the singly encountered 8n × 2 and 16n types [15]. The signals regulating the steady accumulation of polyploid hepatocytes in developing and aging humans remain unknown but may be related to the insulin-AKT pathway [16].

Normally, binucleate cells are potential sources of future clones of mononucleate polyploid cells with unlimited numbers of descendants. Subsequently, during liver regeneration, the hepatocytes divide by complete mitosis. Mitoses without cytokinesis are temporarily excluded; as a result, monopolyploids predominate in the cell population. Thus, hepatocyte polyploidy is one of the mechanisms for the rapid restoration of the hepatocyte pool in the case of liver damage [13].

Currently, there is wide discussion of the presence of three zones within liver lobules, these differing both in their functions and in the activity of their hepatocyte proliferation during regeneration. Hepatocytes located in the periportal zone are claimed to be specialized in gluconeogenesis and β-oxidation, whereas hepatocytes located around the central vein are more important for glycolysis, lipogenesis, and detoxification. Thus, hepatocytes express different genes, depending on their location along the portocentral axis of the hepatic lobule. This conclusion was drawn on the basis of lineage-tracing experiments in mice, in which a population of proliferating and self-renewing hepatocytes in the centrilobular zone, adjacent to the central vein of the lobule, has been recently identified. A number of works have shown that these cells can proliferate twice as fast as the other hepatocytes and replace more than one-third of the mouse liver lobule around the central vein during liver regeneration [17,18]. In addition, a number of authors have shown the zonality of the distribution of monoploid and polyploid cells in liver lobules. Periportal hepatocytes are preferentially diploid with pericentral hepatocytes being polyploid [19,20]. However, such a strict division into zones is quite controversial, especially because a recent study failed to provide evidence of a hepatocyte niche around the central vein responsible for liver homeostasis and did not find a higher proliferation rate of pericentral hepatocytes [21]. Kreutz et al. also revealed the existence of polyploid hepatocytes in both the periportal and pericentral areas [22].

Recent studies have also established the existence of an alternative way to restore the liver by the differentiation of stem cells, called oval cells or hepatic progenitor cells (HPCs), into hepatocytes. The liver progenitor cells are localized in the canals of Hering of the periportal zone of the liver lobules [23]. These cells are committed multipotent progenitor cells capable of differentiating into hepatocytes and cholangiocytes. Activation of the liver progenitor cells occurs when hepatocytes are chronically damaged under exposure to hepatotoxins or in the presence of a viral infection that suppresses their proliferative response [24]. Liver progenitor cell proliferation is dependent on growth factors produced by stellate cells, including HGF, fibroblast growth factors 1 and 2 (FGF1, FGF2) and VEGF. Liver progenitor cells, capable of the production of albumin and alpha-fetoprotein, become basophilic hepatocytes within 4–5 days of activation, and eventually, these cells can become mature hepatocytes [25]. In the rat, there is some circumstantial evidence suggesting that the oval cells can regenerate hepatocytes when hepatocyte proliferation is compromised. However, the contribution of the oval cells to the regeneration process in non-toxic damage is insignificant, even with a loss of up to 80% of the liver volume [26]. Accurate identification of the contribution of oval cells to the regeneration process requires reliable clone tracking systems to provide evidence of regenerative clones in the commonly studied models. Another issue is whether liver damage models reliably reflect the severity of liver damage seen in human diseases. Morphological studies have claimed that HPCs regenerate hepatocytes in areas of liver parenchyma that have been obliterated [27]. However, the analogues of oval cells in humans have not yet been fully defined.

Another alternative regenerative scheme suggests that hepatocytes and cholangiocytes function as ‘facultative stem cells’ for each other. Such a scenario has been shown when blocking hepatocyte proliferation using 2-acetylaminofluorane, a chemical carcinogen that forms DNA adducts. Similar results have also been found in cases of chronic liver injury, when the suppression of hepatocyte proliferation triggered the expansion of progenitor cells, this being known as the ‘ductular reaction’ [28,29]. Select cholangiocyte subpopulations might have an enhanced capacity to function as bipotential progenitor cells and to participate in the process of liver recovery. Bin Li et al. have shown that cholangiocytes in close proximity to hepatocytes at the end of branches of biliary ductules, extending deep into the canals of Hering, have promiscuous expression of both hepatocyte and cholangiocyte transcription factors [29], meaning that new hepatocytes and cholangiocytes can be derived from homotypic precursors [30]. Nonetheless, the precise cells of origin and the regenerative potential of these cells remain contentious to this very day. Evidence supporting biliary ancestry comes from lineage tracing experiments in Sox9-CreER and Opn-CreER mice and from the fact that whole ductal tree fragments or ductal marker-enriched (e.g., EpCAM+, MIC1-1C3+, CD24+, CD133+) single cells self-renew in vitro as 2D monolayers or 3D organoid cultures [31,32] while maintaining potency toward the hepatocyte lineage. However, several recent studies, as in the case of hepatic progenitor cells, have shown minimal regeneration of the hepatocyte parenchyma by ductal-derived progenitors in vivo, in contrast to the robust contribution from hepatocytes themselves [33,34].

**Figure 1:**
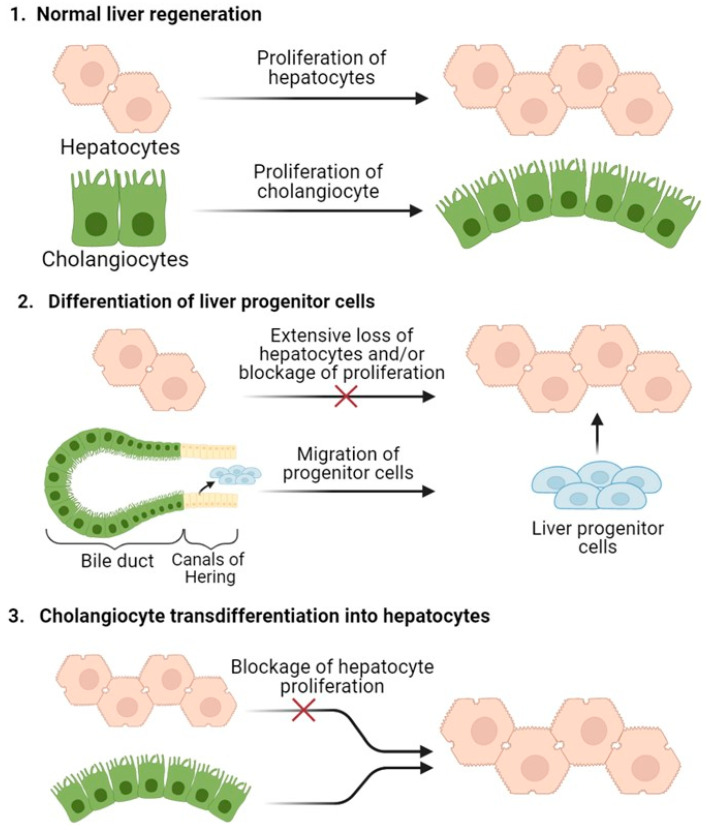
Scheme of the three mechanisms of liver regeneration. For normal regeneration, the mass of the liver is restored to a greater extent by the proliferation of hepatocytes. With extensive damage to hepatocytes or in the case of toxic damage, there is also the possibility of the differentiation of progenitor cells and/or the transdifferentiation of cholangiocytes into hepatocytes.

All three types of liver regeneration mechanisms described above are represented in Figure 1.

Signaling and coordination of the regeneration process occur through various cytokines and growth factors. In a classic experiment with parabiosis, Moolten and Bucher [35] suggested the presence of circulating factors that stimulate and regulate the regenerative processes of the liver. Subsequently, it was determined that the regeneration takes place in three main stages, and that the signaling of the process is carried out mainly through various growth factors and cytokines. 

The modern, dominant point of view is that the three types of pathways encompass the essential circuitry required for liver regeneration: cytokine, growth factor and metabolic networks that link liver function with cell growth and proliferation [36].

**Figure 2:**
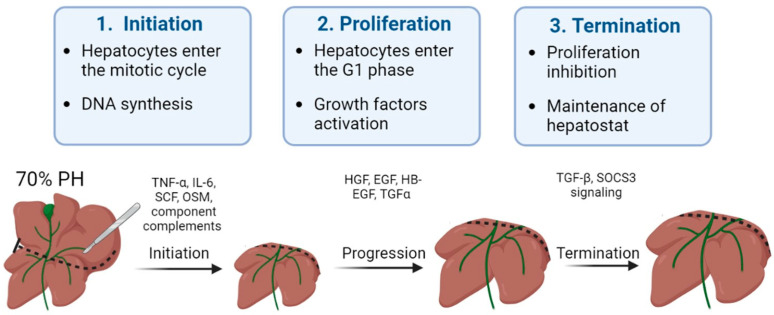
A schematic diagram of the stages and signal molecules involved in liver regeneration.

In the first stage of regeneration, which is called initiation, hepatocytes lose their resting state and enter the mitotic cycle. DNA synthesis starts, triggered by key growth factors including transforming growth factor α (TGF-α), interleykine-6 (IL-6) and, according to some sources, complement components. The duration of the first stage is about 12 h [37]. In the second stage of regeneration, the proliferation phase, hepatocytes enter the G1 phase of the cell cycle, and the mitogens (growth factors) HGF, EGF, heparin-binding epidermal growth factor (HB-EGF) and TGF-α are activated [38]. This stage takes about 48 h, and normally 80–90% of the initial mass of the liver parenchyma is restored. In some cases, the mass of the liver parenchyma at this stage may exceed the initial mass of the liver, but later, by activating cell apoptosis, the size of the organ returns to normal. During the last stage, the regeneration processes are terminated, this stage being necessary to stop the active proliferation and to establish hepatostat [6,39]. The signaling of the last stage is currently not well investigated, but the key role of transforming growth factor β (TGF-β) has been demonstrated [40]. Additionally, a key aspect is regulation of the synthesis of extracellular matrix (ECM), as a result of communication between the hepatocytes and the hepatic stellate cells (HSCs). This communication is regulated in part by integrin-linked kinase (ILK), a protein expressed in both HSCs and hepatocytes. ILK is a hepatocyte growth suppressor and a regulator of hepatocyte differentiation. ILK-knockout mice have enlarged livers and excessive ECM, composed predominantly of collagens that surround each hepatocyte. At the end of liver regeneration in ILK-knockout mice, the already larger liver extends beyond the original 100% pre-partial hepatectomy size up to 158% [30]. Figure 2 shows the three stages in the regenerative process of a healthy liver.

The regenerative process can also be modeled using various hepatotoxins. When injected, a hepatotoxin causing hepatocellular necrosis or apoptosis of liver cells triggers the process of liver regeneration. Toxic liver damage in most studies is modeled using carbon tetrachloride (CCl4) or acetaminophen (APAP), also known as paracetamol. The mechanism of hepatic regeneration in toxic damage does not fundamentally differ from the mechanisms triggered during liver resection. However, recovery occurs differently at a tissue level. The main difference is that most hepatotoxins cause mosaic damage with necrosis of the central or the peripheral areas of the liver lobules. The zone of selective damage is determined by the predominant localization of the toxin-metabolizing enzymes. As a result, it is the remaining intact hepatocytes of the unaffected areas of the liver lobules that will become the sources of proliferating cells [2,6].

## 2. Liver Regeneration in Hepatic Pathology

The most effective treatment for neoplasms localized in the liver (e.g., tumor, metastases) is resection and relies on the high regenerative potential of this organ. It is known that 25% is the minimum amount of hepatic remnant sufficient for adequate recovery in a patient with a morphologically unchanged liver. In the presence of drug-induced damage or hepatic pathology, there is a requirement for the liver remnant to be at least 40% of the initial volume [41]. When the volume of the resected part of the organ exceeds this limit, the liver is not able to restore sufficient function. However, even in cases of the removal of smaller fragments of the liver, there is a risk of fatal, acute post-resection liver failure, which occurs in 5–8% of patients and remains the main cause of mortality in liver surgery [5,41,42,43,44,45].

Below, we consider the main hepatic pathologies that are widespread in patients and describe the principal metabolic mechanisms that impair both liver function and its regenerative capacity.

### 2.1. Non-Alcoholic Fatty Liver Disease

**Figure 3:**
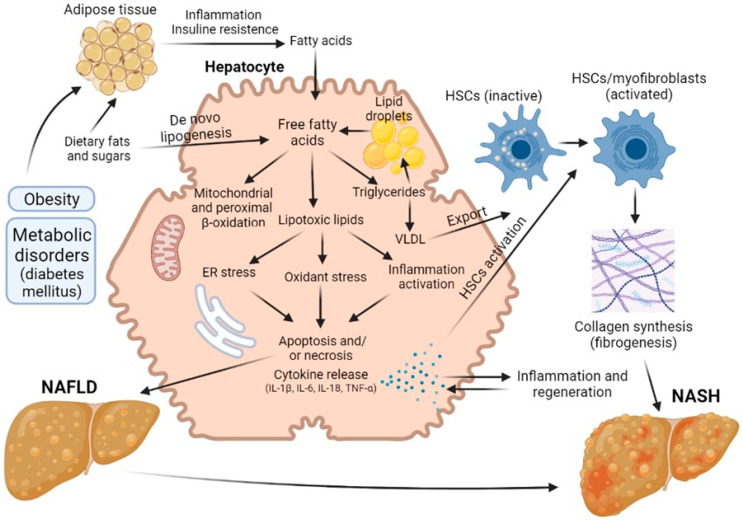
Road map of the pathological mechanisms of NAFLD and NASH.

There has been an explosion of interest in non-alcoholic fatty liver disease (NAFLD) and its more advanced stage, NASH, because of their growing impact on world health. Globally, the prevalence of NAFLD is estimated at 25% of the population, while 25% of NAFLD patients are estimated to have NASH. NAFLD is a general term that includes conditions that vary in the severity of injury and degree of fibrosis. The presence of these pathological conditions dramatically increases the risk of developing cirrhosis and has caused an alarming increase in hepatocellular carcinoma [46,47]. The mechanisms of the pathogenesis of NAFLD and NASH are shown in the Figure 3.

Hepatic steatosis is the most common form of non-alcoholic fatty liver disease and is diagnosed when more than 5% of hepatocytes contain lipid droplets rich in triacylglycerol (TAG) in the absence of a secondary factor such as excessive alcohol intake, viral infection or drug damage [48,49]. Free fatty acids (FFAs) can be obtained from diet, adipose tissue lipolysis and/or de novo lipogenesis. They are then oxidized via β-oxidation, esterified to TAGs and packaged into lipoproteins, which are either secreted or stored as lipid droplets. NASH, as opposed to steatosis, is typically accompanied by pericellular fibrosis, which may progress to cirrhosis [33].

Initially, it was believed that the synthesis of triacylglycerol and the accumulation of fat in the liver was a hepatoprotective mechanism, but later data appeared indicating that such excess content of lipids in the liver cells is a risk factor for the progression of liver disease [48,49]. Steatosis was not diagnosed until 1980, but it is now believed that the presence of steatosis is a risk factor that worsens the prognosis of liver regeneration after resection. Today, about 20% of liver resection patients and up to 25% of liver transplant donors have some degree of steatosis. In a number of studies, it has been demonstrated that the risk of postoperative complications and lethal outcomes grows with increasing severity of steatosis [48,49,50,51,52,53]. In addition, patients with hepatic steatosis are particularly vulnerable to ischemia/reperfusion injury, which can often occur during surgery and liver transplantation [54].

These days, NAFLD, and in particular steatosis, is recognized as a risk factor for resection, although the exact mechanisms of this issue has not yet been established. A number of authors have proposed a sequence known as the “two-hit theory”, which suggests a two-stage process of disease development [55,56]. As stated above, at the first stage, the initial pathological changes are caused by an excess of FFAs in the liver, which are then metabolized to TAG [51,57]. Such processes make the liver vulnerable to aggressive factors at the second stage, caused by oxidative stress and the action of pro-inflammatory cytokines [58]. The ‘two-hit’ theory was posited for several years. However, this view is now considered outdated. There are many molecular pathways that contribute to the development of NAFLD and NASH, and it is not even certain whether NASH is always preceded by NAFLD. In defining the pathogenic drivers of NAFLD and NASH, a useful conceptual framework is that the liver’s capacity to handle the primary metabolic energy substrates, carbohydrates and fatty acids is overwhelmed, leading to the accumulation of toxic lipid species [59,60,61]. These metabolites induce hepatocellular stress, injury and death, leading to fibrogenesis and genomic instability that predispose to cirrhosis and hepatocellular carcinoma. Thus, when fatty acids are either supplied in excess, or their disposal is impaired, they may serve as substrates for the generation of lipotoxic species that provoke oxidative stress and hepatocellular injury.

This chain of events leads to the development of a key factor in the pathogenesis of steatosis—mitochondrial dysfunction [62,63], an event causing a decrease in the regenerative potential of the liver. In a study comparing patients with low (3%) and high (>17%—moderate steatosis) levels of intrahepatic triacylglycerol, it was found that in the presence of steatosis, the rates of lipolysis and of gluconeogenesis increased by 50% and by 30%, respectively. Normally, long-chain fatty acids are metabolized into acyl-CoA molecules by specific acyl-CoA synthases and then enter the mitochondrial matrix. At the same time, reduced NADH, formed as a result of β-oxidation and the operation of the tricarboxylic acid cycle, deliver their electrons and protons to the respiratory chain of mitochondria with the formation of NAD+ and adenosine triphosphate (ATP) [64]. Excessive influx of fatty acids into the liver leads to an overload of the mitochondria, resulting in an accumulation of incompletely oxidized substrates, with increased formation of ROS [65,66]. Chronic oxidative stress leads to the depletion of antioxidants such as glutathione, thereby further compounding cell damage [46]. In addition, persistent oxidative stress leads to disruption of the integrity of the mitochondrial membrane and, therefore, to mitochondrial dysfunction, which is defined by a number of authors as a key event triggering an irreversible cascade of damage to liver cells. As a result, the production of mitochondrial ATP in fatty hepatocytes decreases, and the overall energy metabolism in the cells is disrupted [51,67,68], this representing the main contributory factor in the decrease in regenerative capacity of the liver in the presence of this pathology.

### 2.2. Diabetes-Provided Fatty Liver Disease

Aside from being caused by excessive consumption of dietary fat, fatty liver develops with metabolic disorders—in particular, diabetes mellitus of types I and II. Diabetes is a chronic metabolic disorder with a rapidly increasing prevalence. It alters the carbohydrate, lipid and protein metabolisms of patients. The World Health Organization predicts that 300 million people around the world will suffer from diabetes mellitus by 2025 [69]. 

Diabetes mellitus has the clearest biological link to the progression of NAFLD, and up to 75% of individuals with diabetes have NAFLD; subsequently, 2–3% of patients develop NASH [46,70,71,72]. The development of fatty liver disease, combined with concomitant hypertension, a characteristic of diabetes mellitus, sharply reduces the regenerative potential of the liver. Diabetes mellitus is also considered a risk factor for prognosis after liver resection in patients with hepatocellular carcinoma, and postoperative morbidity is more common among diabetic patients than among nondiabetic patients. Several critical pathways have been identified as causing liver damage in diabetic patients. Insulin resistance, the main cause of hyperglycaemia and compensatory hyperinsulinaemia, is the predominant cause of impaired regenerative response [71,73]. It has been shown that streptozotocin-induced diabetic rats subjected to PH present with a decrease in the level of proliferating cell nuclear antigen and a significant decrease in cyclin D1 levels, suggesting that few hepatocytes are capable of entering the cell cycle [74]. The reasons are that diabetes is generally followed by increased free radical production or reduced antioxidant protection, leading to lipid peroxidation. Such streptozotocin-induced diabetic rats were also found to present with an increase in •OH production, which could result in DNA damage. Hyperglycemia in these rats leads to an increase in hepatic ROS production and is further exacerbated after PH. Oxygen free radicals are extensively formed in diabetic patients by glucose oxidation, non-enzymatic protein glycation and subsequent oxidative degradation of the glycated proteins [71]. Hyperglycemia-induced oxidative stress, followed by derangement of protein, carbohydrate and lipid metabolism, thereby leads to increased oxidative stress and to further triggering of the inflammatory cascade. Both oxidative stress and inflammatory responses act as damaging agents in aggravating the pathological condition of diabetes [75]. Eventually, such violation of the metabolism of liver cells will inevitably disrupt the regenerative capacity of the liver.

### 2.3. Fibrosis and Cirrhosis

Liver fibrosis, or its decompensated form, cirrhosis, develops in response to chronic liver damage of various origins. Fibrosis is characterized by excessive formation and deposition of extracellular matrix and collagen [46], accompanied by a violation of tissue architecture, the development of portal hypertension and cellular hypoxia. The progression of fibrosis results from chronic damage and chronic recovery process in the liver. In cases of acute, rather than severe, liver damage, the remaining mature hepatocytes are able to replace apoptotic and necrotic cells [76], but in chronic damage, the regenerative process is disrupted, and hepatocytes are replaced by extracellular matrix proteins [77,78]. A key event in liver fibrosis is the activation of HSCs, resulting in these cells acquiring a myofibroblast-like phenotype characterized by active proliferation, loss of vitamin A stores and activation of the production and release of alpha-smooth muscle actin and collagen (types I and III) into the extracellular space [79]. In addition, upregulation of a tissue inhibitor of metalloproteinase-1 in the fibrotic liver contributes to collagen deposition by inhibiting the dissolution of the extracellular matrix. Persistent production of growth factors for HSCs, fibrogenic cytokines and chemokines by various types of liver cells is involved in fibrogenesis in chronic inflammation. Among these, TGF-ß, produced by immune cells, directly promotes fibrogenesis by inducing the transcription of types I and III collagen through the Smad signaling pathway [80]. IL-1ß and TNF-α do not induce HSC activation but do contribute to liver fibrosis by, instead, mediating the survival of the activated HSCs [80]. 

**Figure 4:**
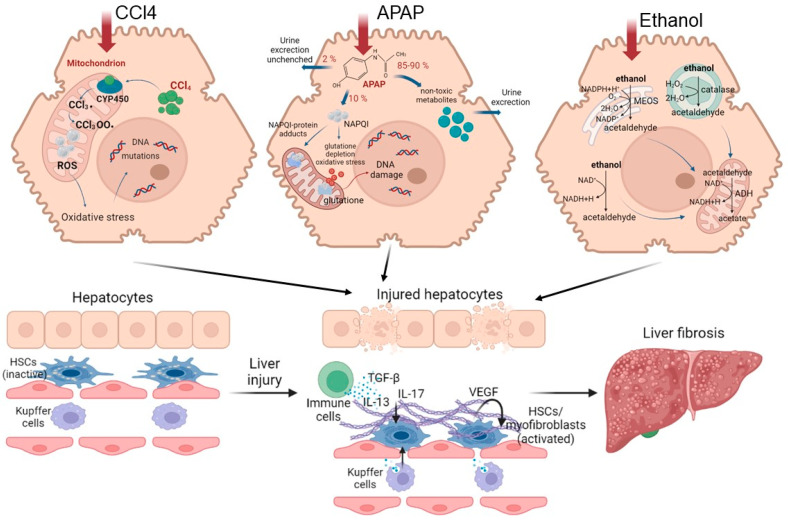
Road map of pathological mechanisms of liver fibrosis when exposed to various damaging agents.

As mentioned earlier, the polyploidy of liver cells plays an important role in ensuring the efficiency of regeneration. However, in chronic diseases that are characterized by compensatory regeneration (in particular, liver cirrhosis), the number of polyploid cells increases by 20%. The frequency of polyploid mononuclear cells and binuclear hepatocytes increases in direct proportion to the severity of fibrosis [15,81]. When chronic loss of hepatocytes and activation of HSCs leads to an abnormal tissue environment, the chronic compensatory regeneration of hepatocytes often has negative consequences, including the development of neoplasia [30]. The mechanisms of the pathogenesis of liver fibrosis are shown in the Figure 4.

Understanding the mechanisms of the pathogenesis of fibrosis has been achieved using various models. CCl4 is widely used to model chronic liver fibrosis and cirrhosis. In hepatocytes, the toxin is metabolized with the participation of cytochrome P450, resulting in the formation of the highly reactive free radicals CCl3• and CCl3O2•. These free radicals activate the fatty acid peroxidation reactions of the mitochondrial membrane and disrupt the integrity and stability of the mitochondrial structure, leading to mitochondrial dysfunction and, ultimately, to necrosis of hepatocytes in the central and parts of the intermediate zones of all liver lobules [82,83,84]. Normally, in the mitochondrial respiration reactions used for energy production, a significant number of electrons are transferred to ATP, while only a few electrons reduced with the formation of ROS. When exposed to CCl4, the structure and functions of the mitochondria are disrupted [85], causing additional generation of ROS. As a result, such a cascade of events leads to the death of hepatocytes by necrosis, thereby affecting liver function. With regular repeated exposure to the toxin, a constant inflammatory and pathological regenerative process is maintained, characterized by the development of chronic regeneration nodes, which ultimately leads to the active accumulation of collagen fibers in response to such chronic damage.

Another commonly used model for the induction of fibrosis and cirrhosis is acetaminophen (APAP) administration. Toxicity due to APAP overdose may arise as a consequence either of an acute overdose or from repeated/staggered dosing over a short period of time. Briefly, APAP hepatotoxicity can be divided into three phases. During the initiation phase after an overdose, APAP is rapidly metabolized to its reactive metabolite, N-acetyl-p-benzoquinone imine (NAPQI), which is removed by glutathione conjugation, leading to rapid depletion of the cellular glutathione stores. The excess NAPQI forms cellular protein adducts, particularly in mitochondria, leading to mitochondrial dysfunction and to the generation of ROS [86]. A previous study utilizing incremental doses of APAP in mice showed that liver regeneration after APAP toxicity was dose dependent. Low overdose of APAP in mice (300 mg/kg) caused not only extensive liver injury but also significant compensatory regeneration, leading to regression of the injury and spontaneous recovery. However, after a severe overdose of APAP (600 mg/kg), liver regeneration was remarkably inhibited, resulting in sustained injury and decreased survival. It is interesting that the marked inhibition of regeneration at the higher dose was not due to a lack of critical liver mass, as >50% of hepatocytes were still viable at this dose, even at peak injury. In fact, peak injury was not remarkably different between the two doses, whereas regeneration was significantly impaired only at the higher dose [87]. The injury phases of APAP hepatotoxicity are subsequently followed by a recovery phase, in which compensatory hepatocellular proliferation is initiated; dead cells are replaced by newly formed cells, leading to liver regeneration and recovery. In cases in which a robust liver-regeneration response is initiated, liver injury is resolved, and liver function is restored spontaneously. In cases in which liver regeneration fails, acute liver injury can progress to acute liver failure, with multi-organ failure and death. Comprehensive analysis of the signaling pathways revealed that several pro- and anti-regenerative pathways were differentially affected in a dose-dependent manner. APAP-stimulated liver injury is characterized by considerably increased expression of IL-1β, IL-18 and the levels of other inflammatory dependent mediators. Paradoxically, dose-dependent activation of EGFR was observed after APAP overdose, such that the activation of EGFR was greater at the higher doses of APAP, where liver regeneration was inhibited. Whereas early inhibition of EGFR activation by pharmacological intervention in mice remarkably attenuated APAP hepatotoxicity, delayed inhibition of EGFR activation led to impaired compensatory liver regeneration, suggesting a dual role of EGFR in both injury initiation and the subsequent liver regeneration after APAP overdose [88]. Elevated plasma levels of inactive, single-chain HGF have also been observed in patients who overdose on APAP. Levels of the angiogenic factor VEGF and expression of its receptors VEGFR1, VEGFR2 and VEGFR3 also increase in mouse liver after paracetamol overdose. The expression of cytokines (TNF and IL-6) also increases after APAP overdose in mice. The foregoing indicates a compensatory activation of pathological recovery processes.

### 2.4. Alcoholic Liver Disease

Another common pathological condition is alcoholic fatty liver disease (ALD), caused by chronic alcohol (ethanol) abuse. A subset of patients with ALD will progress to develop alcohol steatohepatitis and then fibrosis if they continue to consume alcohol heavily [89]. In the later stages, ALD is characterized by a marked fibrotic response and the development of advanced fibrosis, which is associated with early mortality. The pattern of fibrosis in ALD is characterized by pericellular and perisinusoidal (terminal small blood vessels with fenestrated discontinuous epithelium in the liver) matrix accumulation. When fibrosis becomes advanced, the liver becomes cirrhotic and consists predominantly of fibrotic tissue, which leads to a major disturbance of hepatic blood flow due to narrowing of the vascular structures within the hepatic lobules, including the sinusoids. In addition, the function of the liver decreases owing to the loss of hepatocytes [89,90].

This scenario of the development of the disease is due to the specifics of ethanol metabolism in hepatocytes. The oxidative pathways of alcohol metabolism involve three enzymes: alcohol dehydrogenase (ADH) in the cytosol, which converts alcohol to acetaldehyde; at elevated ethanol concentrations, CYP2E1 in the microsomes, which also assumes an important role in metabolizing ethanol to acetaldehyde; while catalase, in the peroxisomes, requires hydrogen peroxide to oxidize alcohol [89]. Acetaldehyde, produced by alcohol oxidation through any of the mechanisms outlined above, is rapidly metabolized to acetate, to form acetate and NADH. The NADH is then oxidized in the mitochondria. Acetaldehyde has the capacity to form protein adducts in hepatocytes, and this has been proposed as an initiator of pathological processes. Structural mitochondrial alterations caused by acetaldehyde lead to functional impairment, including to decreased ATP generation via the respiratory chain, the production of ROS and a decrease in acetaldehyde dehydrogenase activity (an enzyme located in mitochondria that is responsible for the metabolism of acetaldehyde to acetate) [89,90]. These metabolic changes are exacerbated by impaired β-oxidation and decreased very-low-density lipoprotein secretion, promoting lipid infiltration of the hepatocytes. Such irreversible metabolic changes in liver cells impair the ability of the liver to recover.

Violation of the recovery capacity of liver in ALD is also reflected in a decrease in the proliferative activity of hepatocytes. Dippold, R. P. et al. showed that after PH, DNA synthesis was, at 24 h, already drastically inhibited in the livers of ethanol-fed rats compared with pair-fed rats. Delay in proliferation in the ethanol-treated liver is associated with a lack of induction of cell cycle genes after PH [91]. There was also impairment of normal miRNA signaling during the regeneration process. At a cellular level, chronic alcohol consumption induces senescent replication of hepatocytes [92]. Evidence suggests that hepatocyte-specific inhibition of miR-122 is associated with features of ALD in mice, and a combination of alcohol feeding and miR-122 inhibition accelerates alcohol-induced liver injury, steatosis, inflammation and fibrosis [89].

When the disease passes into a chronic form, inflammation is the main factor involved in the progression of ALD. While both ethanol and acetaldehyde are direct hepatotoxins, excessive ROS production and the subsequent production of inflammatory cytokines can promote alcohol-induced liver injury and inflammation. In addition, ethanol and acetaldehyde directly injure hepatocyte mitochondria, upregulating mitochondrial ROS production and further promoting liver injury and inflammation [93]. Damage-associated molecular patterns are evident after cell death—mainly necrosis—and trigger macrophage and neutrophil activation, fibrogenesis and hepatic regeneration [93].

## 3. Future Perspectives

The mechanisms of violation of the regenerative potential of liver with concomitant pathologies have been described above. Next, we consider promising approaches to stimulating liver regeneration.

### 3.1. Promising Approaches to Stimulate Liver Regeneration 

The most promising is the stimulation of regeneration under the action of various bioactive molecules, and there are several approaches to their delivery to liver cells. One such approach is the transplantation of mesenchymal stem cells (MSCs) or the use of their secretome, which contains a wide variety of secreted growth factors, chemokines and hormones, including vascular endothelial growth factor (VEGF) [94,95,96], basic fibroblast growth factor (FGF2), insulin-like growth factor (IGF-1) [96,97], transforming growth factor-beta (TGF-β1), interleukin (IL-6, IL-8) [98], hepatocyte growth factor (HGF) [96,97], tumor necrosis factor alpha (TNF-α) and some other bioactive molecules. On the one hand, this approach, due to its complexity, has already shown its effectiveness in a number of works [99,100,101]. On the other hand, the wide range of possible targets entails a high risk of systemic side effects.

Another approach is the injection of specific growth factors. The most commonly used for these purposes are HGF, EDGF and VEGF. In contrast to the MSC secretome, the advantaged of this approach are the marginal stimulation of cell proliferation and the low risk of systemic exposure. 

An even more targeted approach is based on mRNA incorporated into the protein synthesis machinery of the target cell to induce the expression of a desired protein [102,103].

The use of non-coding miRNAs is also relevant. The regulatory role of some types of miRNAs in the cascade of various molecular pathways during liver regeneration has been established, and about 26 types of miRNAs are known to change their expression levels by more than one and a half times after partial hepatectomy. The most pronounced changes are shown for miRNA-21 [104], miRNA-19a, and miRNA-214 [105]. A number of other miRNAs, such as miRNA-106a, miRNA-20a, miRNA-20b and miRNA-93, have been identified as modulators of VEGF, which acts as a secondary mitogen in hepatocytes during regeneration [106].

However, there is a significant drawback in approaches based on the introduction of naked bioactive molecules. Bioactive molecules have only a short period of circulation and can provoke undesirable side effects. In addition, under the action of enzymes in the bloodstream, they are rapidly degraded or inactivated [107]. Therefore, there remain the problems of their effective delivery to liver cells with a controlled period of release of such molecules, their accumulation and excretion.

The identification of effective, nontoxic delivery systems remains one of the biggest challenges preventing the widespread clinical application of bioactive molecules in therapeutics. Although viral systems such as lentiviruses, adeno-associated viruses and the Sendai virus are capable of systemic nucleic acid delivery, their use can be limited by unwanted immune responses and mutations caused by the inserted sequence [108,109]. In this regard, liposomes and nanoparticles seem to be more promising. A liposome of this type is composed of a membrane-like surface, with the nucleic acids encapsulated inside. Liposomes have unique characteristics including their ease of production, high affinity with the cell membrane plus their nonpathogenic and non-immunogenic nature. The most critical disadvantage of a liposome delivery system is the short half-lives (several hours) of liposomes in blood serum due to nonspecific binding to serum proteins [110]. Polymer nanoparticle approaches utilize polyethylenimine, polylactide (or polylactide-co-glycolide) or polyamidoamine dendrimers as delivery carriers. Such nanoparticles show only a low level of damage to cell membranes and low cytotoxicity. There have also been a few studies of the application of inorganic materials for biomolecule delivery. Current inorganic delivery systems include gold nanoparticles (most frequently used), Fe_3_O_4_-based nanoparticles and silica-based nanoparticles [109]. 

Delivery systems can be modified by attaching additional molecules to enhance their targeting to specific cells. The first step for efficient internalization is the interaction between the delivery system and the cell membrane. The attachment to the cell surface may occur through electrostatic interactions between the system and the membrane surface. Cell binding can also be improved by inclusion in the structure of ligands able to interact with specific cell surface receptors [111,112].

However, it is not enough just to penetrate the cell; it is also important to preserve the bioactive properties of the molecules. The main mechanism of cell entry is endocytosis. The endosomes mature and fuse with lysosomes, where the acidic environment and the presence of hydrolytic enzymes can degrade the biomolecules. Therefore, limitations on endosomal escape before degradation are considered the present bottleneck for successful therapy based on such delivery systems. The foremost proposed mechanisms of endosomal escape include endosome disruption, active transport or the fusion of the delivery system with the endosomal membrane [113,114]. However, these approaches are currently not sufficiently effective, and research in this area is still ongoing.

### 3.2. Prospects for Predictive Liver Assessment

Not least important is the development of new methods for assessing the effectiveness of regeneration and even of conducting predictive assessments of the regenerative potential after liver resection. In clinical practice, the volume and structure of the liver are examined using imaging methods such as ultrasound, CT and MRI [115,116]. However, despite the widespread use of these methods, they have a number of shortcomings that do not allow an adequate assessment of the regenerative potential of the liver. First of all, the density of the liver changes depending on the degree of deposition of lipids, glycogen and non-resident inflammatory cells, which may or may not be associated with regenerative or hyperplastic activity [117]. The disadvantages of CT include the need to introduce a contrast agent and the use of X-rays. MRI does not require radiation, but the introduction of a contrast agent is still necessary [118], and this can limit the application of MRI for patients with various excretory dysfunctions. 

The simplest and most widely used method for the assessment of liver function, and tightly associated with the ability of the liver to recover, is a biochemical blood test. However, this method is not specific, reflecting disorders not only of the liver but also of the body as a whole [119,120]. Furthermore, the elimination period of standard markers is too long to reflect pathological changes in real time [121,122]. More effective are functional tests evaluating the rate of excretion (clearance) by the liver of various exogenous substances. The most common is the clearance of indocyanine green [123]. Clearance tests allow for predicting complications and the survival of patients after liver resection with a certain degree of probability. However, it is possible to assess liver function only at the time of surgery, without a predictive assessment of the hepatic remnant [124]. As in the case of MRI, the use of clearance tests is limited in patients with excretory disorders due to the need to administer a contrast agent [125].

Thus, the search continues for new approaches to assessing hepatic function with the possibility of predicting the regenerative capacity of the liver, primarily taking into account concomitant pathology. Multiphoton microscopy with second harmonic generation (SHG) modalities, used in association with fluorescent life-time imaging microscopy (FLIM), may become such an approach. Multiphoton microscopy visualizes the structure of the liver tissue and makes it possible to identify areas of damaged cells by the intensity of autofluorescence of endogenous cofactors, in particular flavine adenine dinucleotide (FAD) and nicotinamide adenine dinucleotide (NADH) [126]. SHG provides additional information about the accumulation of collagen in the tissue, which is critical for diagnosing the stage of hepatic pathology [127]. Thus, the combination of these two methods becomes analogous to real-time histology [128]. However, this is not all; using FLIM can also provide a predictive assessment of the state of the liver tissue. The vast majority of FLIM studies are based on the estimation of NADH fluorescence lifetimes. This cofactor is well studied, given its participation in many key metabolic pathways, including the tricarboxylic acid cycle, oxidative phosphorylation (OXPHOS) and glycolysis [129]. Under normal conditions, most of the ATP in liver cells is produced by the chain of reactions known as the mitochondrial OXPHOS system, and the bound form of NADH is involved in this process. The second pathway for ATP synthesis is glycolysis, where a free form of NADH is involved. However, the contribution of glycolysis to the energy metabolism of metabolically active cells is insignificant [130]. FLIM makes it possible to estimate the intensities of OXPHOS and glycolysis based on data on the fluorescence lifetimes of the free and bound forms of the NADH. A shift of the metabolic state in one direction or the other indicates the presence of pathological rearrangements in cells. As described in detail earlier, there are various pathological conditions associated with mechanisms that disrupt the native metabolism of liver cells. Thus, a change in the metabolic state of cells can be considered a sensitive marker of the function of the entire organ. In addition, the use of machine learning methods to automate the processing of data arrays obtained by FLIM will bring this approach closer to the clinic [131,132].

Finally, a promising approach is the development of computer models that will allow predictive assessment of the dynamics of the liver response to resection. In particular, Verma et al. analyzed the responses to resection with individual parameter variations for different “virtual patients” based on their clinical tests and the effect on the regenerative outcome. They revealed that the response mode and extent of recovery following resection were most sensitive to variations in two perioperative factors, increased metabolic load on hepatocytes and cell death post partial hepatectomy. Increase in metabolic load per unit of liver mass following PHx (due to the reduced remnant liver mass) elicits a cascade of signals, leading to the activation of hepatic non-parenchymal cells, which in turn release cytokines and various growth factors, targeting signaling pathways in the hepatocytes. This results in the progression of hepatocytes through the cell cycle [133,134]. Such computer model-based approaches have the potential for predictive assessment based on patient characteristics [135].

## 4. Conclusions and Outlooks

This review has described the main known mechanisms that reduce the regenerative potential of liver with the most common liver pathologies. The key aspects are impaired hepatocyte metabolism and lack of signaling for the regenerative process. Despite the different origins of the diseases described, the main metabolic mechanisms are similar, the key factor being oxidative stress, which leads to disruption of the integrity of the cell structure or the mitochondrial membrane. In turn, disruption of mitochondrial integrity triggers a vicious cycle leading to mitochondrial dysfunction and, ultimately, to death of the liver cells. Next, two scenarios follow. In the first case, activation of compensatory pathological restoration of the liver tissue occurs, due to both the proliferation of hepatocytes (or, in some cases, of progenitor liver cells) and the growth of the ECM (fibrogenesis). These events lead to the development of fibrosis or even neoplasia. In the second case, the liver’s ability to recover is irreversibly compromised, resulting in liver failure, a serious condition with a high lethal risk. Understanding the mechanisms that underlie not only the normal but also the violated regenerative processes could enhance therapeutic opportunities.

Due to the wide spread of concomitant hepatic pathologies, the question arises in the search for new approaches of how to ensure appropriate liver regeneration. The main challenge is the targeted delivery of regeneration-stimulating molecules to specific cells while maintaining the molecules’ bioactive properties. It is also important to develop modern methods for assessing the state of the liver. Furthermore, new clinical diagnostic methods need to be based not only on assessment of the liver volume and its tissue structure but also on the provision of predictive assessments of the regeneration potential of the liver remnant. It is evident that the development of new methods for assessing the regenerative potential of liver remnants and of strategies to stimulate such regeneration should not underestimate pathological changes at the level of cell metabolism.

## Data Availability

Not applicable.

## References

[1] Michalopoulos G.K., Arias I.M., Alter H.J. (2020). The Liver: Biology and Pathobiology.

[2] Michalopoulos G.K. (2017). Hepatostat: Liver regeneration and normal liver tissue maintenance. Hepatology.

[3] Lu J., Zhao Y.L., Zhang X.Q., Li L.J. (2021). The vascular endothelial growth factor signaling pathway regulates liver sinusoidal endothelial cells during liver regeneration after partial hepatectomy. Expert Rev. Gastroenterol. Hepatol..

[4] Higgins G.M. (1931). Experimental pathology of the liver. Restoration of the liver of the white rat following partial surgical removal. Arch. Pathol. Lab. Med..

[5] Bhushan B., Walesky C., Manley M., Gallagher T., Borude P., Edwards G., Monga P.S., Apte U. (2014). Pro-regenerative signaling after acetaminophen-induced acute liver injury in mice identified using a novel incremental dose model. Am. J. Pathol..

[6] Forbes S.J., Newsome P.N. (2016). Liver regeneration—Mechanisms and models to clinical application. Nat. Rev. Gastroenterol. Hepatol..

[7] Miyaoka Y., Ebato K., Kato H., Arakawa S., Shimizu S., Miyajima A. (2012). Hypertrophy and unconventional cell division of hepatocytes underlie liver regeneration. Curr. Biol..

[8] Bangru S., Arif W., Seimetz J., Bhate A., Chen J., Rashan E.H., Carstens R.P., Anakk S., Kalsotra A. (2018). Alternative splicing rewires Hippo signaling pathway in hepatocytes to promote liver regeneration. Nat. Struct. Mol. Biol..

[9] Abu Rmilah A., Zhou W., Nelson E., Lin L., Amiot B., Nyberg S.L. (2019). Understanding the marvels behind liver regeneration. Wiley Interdiscip. Rev. Dev. Biol..

[10] Rio Bartulos C., Senk K., Schumacher M., Plath J., Kaiser N., Bade R., Woetzel J., Wiggermann P. (2022). Assessment of Liver Function With MRI: Where Do We Stand?. Front. Med..

[11] Gentric G., Desdouets C. (2014). Polyploidization in liver tissue. Am. J. Pathol..

[12] Wilkinson P.D., Alencastro F., Delgado E.R., Leek M.P., Weirich M.P., Otero P.A., Roy N., Brown W.K., Oertel M., Duncan A.W. (2019). Polyploid hepatocytes facilitate adaptation and regeneration to chronic liver injury. Am. J. Pathol..

[13] Pandit S.K., Westendorp B., de Bruin A. (2013). Physiological significance of polyploidization in mammalian cells. Trends Cell Biol..

[14] Gentric G., Celton-Morizur S., Desdouets C. (2012). Polyploidy and liver proliferation. Clin. Res. Hepatol. Gastroenterol..

[15] Donne R., Saroul-Aïnama M., Cordier P., Celton-Morizur S., Desdouets C. (2020). Polyploidy in liver development, homeostasis and disease. Nat. Rev. Gastroenterol. Hepat..

[16] Delgado E.R., Stahl E.C., Roy N., Wilkinson P.D., Duncan A.W. (2020). The Liver: Biology and Pathobiology.

[17] Wang B., Zhao L., Fish M., Logan C.Y., Nusse R. (2015). Self-renewing diploid Axin2^+^ cells fuel homeostatic renewal of the liver. Nature.

[18] Font-Burgada J., Shalapour S., Ramaswamy S., Hsueh B., Rossell D., Umemura A., Taniguchi K., Nakagawa H., Valasek M.A., Ye L. (2015). Hybrid periportal hepatocytes regenerate the injured liver without giving rise to cancer. Cell.

[19] Gandillet A., Alexandre E., Holl V., Royer C., Bischoff P., Cinqualbre J., Wolf P., Jaeck D., Richert L. (2003). Hepatocyte ploidy in normal young rat. Comp. Biochem. Physiol. Part A Mol. Integr. Physiol..

[20] Asahina K., Teramoto K., Teraoka H. (2006). Embryonic stem cells: Hepatic differentiation and regenerative medicine for the treatment of liver disease. Curr. Stem Cell Res. Ther..

[21] Planas-Paz L., Orsini V., Boulter L., Calabrese D., Pikiolek M., Nigsch F., Xie Y., Roma G., Donovan A., Marti P. (2016). The RSPO–LGR4/5–ZNRF3/RNF43 module controls liver zonation and size. Nat. Cell Biol..

[22] Kreutz C., MacNelly S., Follo M., Wäldin A., Binninger-Lacour P., Timmer J., Bartolomé-Rodríguez M.M. (2017). Hepatocyte ploidy is a diversity factor for liver homeostasis. Front. Physiol..

[23] Michalopoulos G.K., Khan Z. (2015). Liver stem cells: Experimental findings and implications for human liver disease. Gastroenterology.

[24] Tanaka M., Itoh T., Tanimizu N., Miyajima A. (2011). Liver stem/progenitor cells: Their characteristics and regulatory mechanisms. J. Biochem..

[25] Mao S.A., Glorioso J.M., Nyberg S.L. (2014). Liver regeneration. Transl. Res..

[26] Dusabineza A.C., Hul N.K.V., Abarca-Quinones J., Starkel P., Najimi M., Leclercq I.A. (2012). Participation of liver progenitor cells in liver regeneration: Lack of evidence in the AAF/PH rat model. Lab. Investig..

[27] Stueck A.E., Wanless I.R. (2015). Hepatocyte buds derived from progenitor cells repopulate regions of parenchymal extinction in human cirrhosis. Hepatology.

[28] Raven A., Lu W.Y., Man T.Y., Ferreira-Gonzalez S., O’Duibhir E., Dwyer B.J., Thomson J.P., Meehan R.R., Bogorad R., Koteliansky V. (2017). Cholangiocytes act as facultative liver stem cells during impaired hepatocyte regeneration. Nature.

[29] Li B., Dorrell C., Canaday P.S., Pelz C., Haft A., Finegold M., Grompe M. (2017). Adult mouse liver contains two distinct populations of cholangiocytes. Stem Cell Rep..

[30] Michalopoulos G.K., Bhushan B. (2021). Liver regeneration: Biological and pathological mechanisms and implications. Nat. Rev. Gastroenterol. Hepat..

[31] Huch M., Gehart H., Van Boxtel R., Hamer K., Blokzijl F., Verstegen M.M., Ellis E., Wenum M., Fuchs S.A., de Ligt J. (2015). Long-term culture of genome-stable bipotent stem cells from adult human liver. Cell.

[32] Lu W.Y., Bird T.G., Boulter L., Tsuchiya A., Cole A.M., Hay T., Guest R.V., Wojtacha D., Man T.Y., Mackinnon A. (2015). Hepatic progenitor cells of biliary origin with liver repopulation capacity. Nat. Cell Biol..

[33] Schaub J.R., Malato Y., Gormond C., Willenbring H. (2014). Evidence against a stem cell origin of new hepatocytes in a common mouse model of chronic liver injury. Cell Rep..

[34] Yanger K., Knigin D., Zong Y., Maggs L., Gu G., Akiyama H., Pikarsky E., Stanger B.Z. (2014). Adult hepatocytes are generated by self-duplication rather than stem cell differentiation. Cell Stem Cell.

[35] Butcher R.L. (1966). Factors affecting luteal regulation following parabiosis in the rat. Endocrinology.

[36] Fausto N., Campbell J.S., Riehle K.J. (2006). Liver regeneration. Hepatology.

[37] Böhm F., Köhler U.A., Speicher T., Werner S. (2010). Regulation of liver regeneration by growth factors and cytokines. EMBO Mol. Med..

[38] Fajardo-Puerta A.B., Prado M.M., Frampton A.E., Jiao L.R. (2016). Gene of the month: HGF. J. Clin. Pathol..

[39] Tao Y., Wang M., Chen E., Tang H. (2017). Liver regeneration: Analysis of the main relevant signaling molecules. Mediat. Inflamm..

[40] Paranjpe S., Bowen W.C., Mars W.M., Orr A., Haynes M.M., DeFrances M.C., Liu S., Tseng G.C., Tsagianni A., Michalopoulos G.K. (2016). Combined systemic elimination of MET and EGFR signaling completely abolishes liver regeneration and leads to liver decompensation. Hepatology.

[41] Guglielmi A., Ruzzenente A., Conci S., Valdegamberi A., Iacono C. (2012). How much remnant is enough in liver resection?. Digest. Surg..

[42] Golse N., Bucur P.O., Adam R., Castaing D., Sa Cunha A., Vibert E. (2013). New paradigms in post-hepatectomy liver failure. J. Gastrointest. Surg..

[43] Nilsson H., Karlgren S., Blomqvist L., Jonas E. (2015). The inhomogeneous distribution of liver function: Possible impact on the prediction of post-operative remnant liver function. Hpb.

[44] Truant S., Scatton O., Dokmak S., Regimbeau J.M., Lucidi V., Laurent A., Gauzolino R., Castro Benitez C., Pequignot A., Donckier V. (2015). Associating liver partition and portal vein ligation for staged hepatectomy (ALPPS): Impact of the inter-stages course on morbi-mortality and implications for management. Eur. J. Surg. Oncol..

[45] Moris D., Vernadakis S., Papalampros A., Vailas M., Dimitrokallis N., Petrou A., Dimitroulis D. (2016). Mechanistic insights of rapid liver regeneration after associating liver partition and portal vein ligation for stage hepatectomy. World J. Gastroenterol..

[46] Friedman S.L., Neuschwander-Tetri B.A., Rinella M., Sanyal A.J. (2018). Mechanisms of NAFLD development and therapeutic strategies. Nat. Med..

[47] Peng C., Stewart A.G., Woodman O.L., Ritchie R.H., Qin C.X. (2020). Non-alcoholic steatohepatitis: A review of its mechanism, models and medical treatments. Front. Pharmacol..

[48] Hamano M., Ezaki H., Kiso S., Furuta K., Egawa M., Kizu T., Chatani N., Kamada Y., Yoshida Y., Takehara T. (2014). Lipid overloading during liver regeneration causes delayed hepatocyte DNA replication by increasing ER stress in mice with simple hepatic steatosis. J. Gastroenterol..

[49] Allaire M., Gilgenkrantz H. (2020). The aged liver: Beyond cellular senescence. Clin. Res. Hepatol. Gastroenterol..

[50] Brunt E.M. (2017). Nonalcoholic fatty liver disease and the ongoing role of liver biopsy evaluation. Hepatol. Commun..

[51] Veteläinen R., van Vliet A., Gouma D.J., van Gulik T.M. (2007). Steatosis as a risk factor in liver surgery. Ann. Surg..

[52] De Meijer V.E., Kalish B.T., Puder M., IJzermans J.N.M. (2010). Systematic review and meta-analysis of steatosis as a risk factor in major hepatic resection. J. Br. Surg..

[53] Kele P.G., van der Jagt E.J., Gouw A.S., Lisman T., Porte R.J., de Boer M.T. (2013). The impact of hepatic steatosis on liver regeneration after partial hepatectomy. Liver Int..

[54] Chu M.J., Hickey A.J., Phillips A.R., Bartlett A.S. (2013). The impact of hepatic steatosis on hepatic ischemia-reperfusion injury in experimental studies: A systematic review. BioMed Res. Int..

[55] Basaranoglu M., Neuschwander-Tetri B.A. (2006). Nonalcoholic fatty liver disease: Clinical features and pathogenesis. Gastroenterol. Hepatol..

[56] Krawczyk M., Bonfrate L., Portincasa P. (2010). Nonalcoholic fatty liver disease. Best Pract. Res. Clin. Gastroenterol..

[57] Michalopoulos G.K. (2010). Liver regeneration after partial hepatectomy: Critical analysis of mechanistic dilemmas. Am. J. Pathol..

[58] Hijona E., Hijona L., Arenas J.I., Bujanda L. (2010). Inflammatory mediators of hepatic steatosis. Mediat. Inflamm..

[59] Cusi K. (2012). Role of obesity and lipotoxicity in the development of nonalcoholic steatohepatitis: Pathophysiology and clinical implications. Gastroenterology.

[60] Hirsova P., Ibrahim S.H., Gores G.J., Malhi H. (2016). Lipotoxic lethal and sublethal stress signaling in hepatocytes: Relevance to NASH pathogenesis. J. Lipid Res..

[61] Mota M., Banini B.A., Cazanave S.C., Sanyal A.J. (2016). Molecular mechanisms of lipotoxicity and glucotoxicity in nonalcoholic fatty liver disease. Metabolism.

[62] Wei Y., Rector R.S., Thyfault J.P., Ibdah J.A. (2008). Nonalcoholic fatty liver disease and mitochondrial dysfunction. World J. Gastroenterol..

[63] Van Zutphen T., Ciapaite J., Bloks V.W., Ackereley C., Gerding A., Jurdzinski A., Moraes R.A., Zhang L., Wolters J.C., Bischoff R. (2016). Malnutrition-associated liver steatosis and ATP depletion is caused by peroxisomal and mitochondrial dysfunction. J. Hepatol..

[64] Ibdah J.A., Bennett M.J., Rinaldo P., Zhao Y., Gibson B., Sims H.F., Strauss A.W. (1999). A fetal fatty-acid oxidation disorder as a cause of liver disease in pregnant women. N. Engl. J. Med..

[65] Cheng Z., Ristow M. (2013). Mitochondria and metabolic homeostasis. Antioxid. Redox Signal..

[66] Koves T.R., Ussher J.R., Noland R.C., Slentz D., Mosedale M., Ilkayeva O. (2008). Mitochondrial overload and incomplete fatty acid oxidation contribute to skeletal muscle insulin resistance. Cell Metab..

[67] Nassir F., Rector R.S., Hammoud G.M., Ibdah J.A. (2015). Pathogenesis and prevention of hepatic steatosis. Gastroenterol. Hepatol..

[68] Marsman H.A., De Graaf W., Heger M., Van Golen R.F., Ten Kate F.J.W., Bennink R., Van Gulik T.M. (2013). Hepatic regeneration and functional recovery following partial liver resection in an experimental model of hepatic steatosis treated with omega-3 fatty acids. J. Br. Surg..

[69] Gargouri M., Magné C., El Feki A. (2016). Hyperglycemia, oxidative stress, liver damage and dysfunction in alloxan-induced diabetic rat are prevented by Spirulina supplementation. Nutr. Res..

[70] Gilgenkrantz H., de l’Hortet A.C. (2018). Understanding liver regeneration: From mechanisms to regenerative medicine. Am. J. Pathol..

[71] Sarin S.K., Choudhury A. (2016). Acute-on-chronic liver failure: Terminology, mechanisms and management. Nat. Rev. Gastroenterol. Hepat..

[72] Lucchesi A.N., Freitas N.T.D., Cassettari L.L., Marques S.F.G., Spadella C.T. (2013). Diabetes mellitus triggers oxidative stress in the liver of alloxan-treated rats: A mechanism for diabetic chronic liver disease. Acta Cir. Bras..

[73] Francés D.E., Ronco M.T., Ingaramo P.I., Monti J.A., Pisani G.B., Parody J., Pellegrino J., Carrillo M.C., Martín-Sanz P., Carnovale C.E. (2011). Role of reactive oxygen species in the early stages of liver regeneration in streptozotocin-induced diabetic rats. Free Radic. Res..

[74] Han Y., Sun T., Han Y., Lin L., Liu C., Liu J., Yan G., Human R.T. (2019). umbilical cord mesenchymal stem cells implantation accelerates cutaneous wound healing in diabetic rats via the Wnt signaling pathway. Eur. J. Med. Res..

[75] Huang X.L., He Y., Ji L.L., Wang K.Y., Wang Y.L., Chen D.F., Geng Y., OuYang P., Lai W.M. (2017). Hepatoprotective potential of isoquercitrin against type 2 diabetes-induced hepatic injury in rats. Oncotarget.

[76] Bataller R., Brenner D.A. (2005). Liver fibrosis. J. Clin. Investig..

[77] Karsdal M.A., Manon-Jensen T., Genovese F., Kristensen J.H., Nielsen M.J., Sand J.M., Hansen N.U.B., Bay-Jensen A.C., Bager C.L., Krag A. (2015). Novel insights into the function and dynamics of extracellular matrix in liver fibrosis. Am. J. Physiol.-Gastrointest. Liver Physiol..

[78] Liang S., Kisseleva T., Brenner D.A. (2016). The role of NADPH oxidases (NOXs) in liver fibrosis and the activation of myofibroblasts. Front. Physiol..

[79] Crespo Yanguas S., Cogliati B., Willebrords J., Maes M., Colle I., Van den Bossche B., Souza de Oliveira C.P.M., Andraus W., Alves V.A., Leclercq I. (2016). Experimental models of liver fibrosis. Arch. Toxicol..

[80] Tanaka M., Miyajima A. (2016). Liver regeneration and fibrosis after inflammation. Inflamm. Regen..

[81] Dewhurst M.R., Ow J.R., Zafer G., van Hul N.K., Wollmann H., Bisteau X., Brough D., Choi H., Kaldis P. (2020). Loss of hepatocyte cell division leads to liver inflammation and fibrosis. PLoS Genet..

[82] Dahlke M.H., Popp F.C., Bahlmann F.H., Aselmann H., Jäger M.D., Neipp M., Piso P., Klempnauer J., Schlitt H.J. (2003). Liver regeneration in a retrorsine/CCl4–induced acute liver failure model: Do bone marrow-derived cells contribute?. J. Hepatol..

[83] Hafez M.M., Al-Shabanah O.A., Al-Harbi N.O., Al-Harbi M.M., Al-Rejaie S.S., Alsurayea S.M., Sayed-Ahmed M.M. (2014). Association between paraoxonases gene expression and oxidative stress in hepatotoxicity induced by CCl4. Oxidative Med. Cell. Longev..

[84] Wang S., Pacher P., De Lisle R.C., Huang H., Ding W.X. (2016). A mechanistic review of cell death in alcohol-induced liver injury. Alcohol. Clin. Exp. Res..

[85] Song M., Yi X., Chen W., Yuan Y., Zhang X., Li J., Tong M., Liu G., You S., Kong X. (2011). Augmenter of liver regeneration (ALR) gene therapy attenuates CCl4-induced liver injury and fibrosis in rats. Biochem. Biophys. Res. Commun..

[86] Ulger O., Kubat G.B., Cicek Z., Celik E., Atalay O., Suvay S., Ozler M. (2021). The effects of mitochondrial transplantation in acetaminophen-induced liver toxicity in rats. Life Sci..

[87] Bhushan B., Apte U. (2019). Liver regeneration after acetaminophen hepatotoxicity: Mechanisms and therapeutic opportunities. Am. J. Pathol..

[88] Bhushan B., Chavan H., Borude P., Xie Y., Du K., McGill M.R., Lebofsky M., Jaeschke H., Krishnamurthy P., Apte U. (2017). Dual role of epidermal growth factor receptor in liver injury and regeneration after acetaminophen overdose in mice. Toxicol. Sci..

[89] Seitz H.K., Bataller R., Cortez-Pinto H., Gao B., Gual A., Lackner C., Mathurin P., Mueller S., Szabo G., Tsukamoto H. (2018). Alcoholic liver disease. Nat. Rev. Dis. Prim..

[90] Barr T., Helms C., Grant K., Messaoudi I. (2016). Opposing effects of alcohol on the immune system. Prog. Neuro-Psychopharmacol. Biol. Psychiatry.

[91] Dippold R.P., Vadigepalli R., Gonye G.E., Patra B., Hoek J.B. (2013). Chronic Ethanol Feeding Alters miRNA Expression Dynamics During Liver Regeneration. Alcohol. Clin. Exp. Res..

[92] Louvet A. (2015). Mathurin, Alcoholic liver disease: Mechanisms of injury and targeted treatment. Nat. Rev. Gastroenterol. Hepatol..

[93] Ohashi K., Pimienta M., Seki E. (2018). Alcoholic liver disease: A current molecular and clinical perspective. Liver Res..

[94] Cooper D.R., Wang C., Patel R., Trujillo A., Patel N.A., Prather J., Gould L.J., Wu M.H. (2018). Human adipose-derived stem cell conditioned media and exosomes containing MALAT1 promote human dermal fibroblast migration and ischemic wound healing. Adv. Wound Care.

[95] Teixeira F.G., Carvalho M.M., Panchalingam K.M., Rodrigues A.J., Mendes-Pinheiro B., Anjo S., Manadas B., Behie L.A., Sousa N., Salgado A.J. (2017). Impact of the secretome of human mesenchymal stem cells on brain structure and animal behavior in a rat model of Parkinson’s disease. Stem Cells Transl. Med..

[96] Togel F., Weiss K., Yang Y., Hu Z., Zhang P., Westenfelder C. (2007). Vasculotropic, paracrine actions of infused mesenchymal stem cells are important to the recovery from acute kidney injury. Am. J. Physiol.-Ren. Physiol..

[97] Inukai T., Katagiri W., Yoshimi R., Osugi M., Kawai T., Hibi H., Ueda M. (2013). Novel application of stem cell-derived factors for periodontal regeneration. Biochem. Biophys. Res. Commun..

[98] Heo S.C., Jeon E.S., Lee I.H., Kim H.S., Kim M.B., Kim J.H. (2011). Tumor necrosis factor-α-activated human adipose tissue–derived mesenchymal stem cells accelerate cutaneous wound healing through paracrine mechanisms. J. Investig. Dermatol..

[99] Hu C., Zhao L., Wu Z., Li L. (2020). Transplantation of mesenchymal stem cells and their derivatives effectively promotes liver regeneration to attenuate acetaminophen-induced liver injury. Stem Cell Res..

[100] Nazarie S.R., Gharbia S., Hermenean A., Dinescu S., Costache M. (2021). Regenerative potential of mesenchymal stem cells’(MSCs) secretome for liver fibrosis therapies. Int. J. Mol. Sci..

[101] Driscoll J., Patel T. (2019). The mesenchymal stem cell secretome as an acellular regenerative therapy for liver disease. J. Gastroenterol..

[102] Gómez-Aguado I., Rodríguez-Castejón J., Vicente-Pascual M., Rodríguez-Gascón A., Solinís M.Á., del Pozo-Rodríguez A. (2020). Nanomedicines to deliver mRNA: State of the art and future perspectives. Nanomaterials.

[103] Kowalski P.S., Rudra A., Miao L., Anderson D.G. (2019). Delivering the messenger: Advances in technologies for therapeutic mRNA delivery. Mol. Ther..

[104] Song G., Sharma A.D., Roll G.R., Ng R., Lee A.Y., Blelloch R.H., Frandsen N.M., Willenbring H. (2010). MicroRNAs control hepatocyte proliferation during liver regeneration. Hepatology.

[105] Cui L., Shi Y., Zhou X., Wang X., Wang J., Lan Y., Wang M., Zheng L., Li H., Wu Q. (2013). A set of microRNAs mediate direct conversion of human umbilical cord lining-derived mesenchymal stem cells into hepatocytes. Cell Death Dis..

[106] Yi P.S., Zhang M., Xu M.Q. (2016). Role of microRNA in liver regeneration. Hepatobiliary Pancreat. Dis. Int..

[107] Lee S.W.L., Paoletti C., Campisi M., Osaki T., Adriani G., Kamm R.D., Mattu C., Chiono V. (2019). MicroRNA delivery through nanoparticles. J. Control. Release.

[108] Hajj K.A., Whitehead K.A. (2017). Tools for translation: Non-viral materials for therapeutic mRNA delivery. Nat. Rev. Mat..

[109] Yang N. (2015). An overview of viral and nonviral delivery systems for microRNA. Int. J. Ppharm. Investig..

[110] Monteiro N., Martins A., Reis R.L., Neves N.M. (2014). Liposomes in tissue engineering and regenerative medicine. J. R. Soc. Interface.

[111] Gan L., Wang J., Zhao Y., Chen D., Zhu C., Liu J., Gan Y. (2013). Hyaluronan-modified core-shell liponanoparticles targeting CD44-positive retinal pigment epithelium cells via intravitreal injection. Biomaterials.

[112] Apaolaza P.S., del Pozo-Rodríguez A., Solinís M.A., Rodríguez J.M., Friedrich U., Torrecilla J., Weber B.H.F., Rodríguez-Gascón A. (2016). Structural recovery of the retina in a retinoschisin-deficient mouse after gene replacement therapy by solid lipid nanoparticles. Biomaterials.

[113] Stewart M.P., Sharei A., Ding X., Sahay G., Langer R., Jensen K.F. (2016). In vitro and ex vivo strategies for intracellular delivery. Nature.

[114] Smith S.A., Selby L.I., Johnston A.P., Such G.K. (2018). The endosomal escape of nanoparticles: Toward more efficient cellular delivery. Bioconjugate Chem..

[115] McNaughton D.A., Abu-Yousef M.M. (2011). Doppler US of the liver made simple. Radiographics.

[116] Will O.M., Damm T., Campbell G.M., von Schönfells W., Açil Y., Will M., Chalaris-Rissmann A., Ayna M., Drucker C., Gluer C.C. (2017). Longitudinal micro-computed tomography monitoring of progressive liver regeneration in a mouse model of partial hepatectomy. Lab. Anim..

[117] Assy N., Minuk G.Y. (1997). Liver regeneration: Methods for monitoring and their applications. J. Hepatol..

[118] Di Martino M., Koryukova K., Bezzi M., Catalano C. (2017). Imaging features of non-alcoholic fatty liver disease in children and adolescents. Children.

[119] Hoekstra L.T., de Graaf W., Nibourg G.A., Heger M., Bennink R.J., Stieger B., van Gulik T.M. (2013). Physiological and biochemical basis of clinical liver function tests: A review. Ann. Surg..

[120] Stockmann M., Lock J.F., Malinowski M., Niehues S.M., Seehofer D., Neuhaus P. (2010). The LiMAx test: A new liver function test for predicting postoperative outcome in liver surgery. Hpb.

[121] Thomas M.N., Weninger E., Angele M., Bösch F., Pratschke S., Andrassy J., Rentsch M., Stangl M., Hartwig W., Werner J. (2015). Intraoperative simulation of remnant liver function during anatomic liver resection with indocyanine green clearance (LiMON) measurements. Hpb.

[122] Wei W., Dirsch O., Mclean A.L., Zafarnia S., Schwier M., Dahmen U. (2015). Rodent models and imaging techniques to study liver regeneration. Eur. Surg. Res..

[123] Helmke S., Colmenero J., Everson G.T. (2015). Non-invasive assessment of liver function. Curr. Opin. Gastroenterol..

[124] Iimuro Y. (2017). ICG clearance test and 99mTc-GSA SPECT/CT fusion images. Visc. Med..

[125] Sumiyoshi T., Shima Y., Okabayashi T., Noda Y., Hata Y., Murata Y., Kozuki A., Tokumaru T., Nakamura T., Uka K. (2014). Functional discrepancy between two liver lobes after hemilobe biliary drainage in patients with jaundice and bile duct cancer: An appraisal using 99mTc-GSA SPECT/CT fusion imaging. Radiology.

[126] Skala M.C., Riching K.M., Gendron-Fitzpatrick A., Eickhoff J., Eliceiri K.W., White J.G., Ramanujam N. (2007). In vivo multiphoton microscopy of NADH and FAD redox states, fluorescence lifetimes, and cellular morphology in precancerous epithelia. Proc. Natl. Acad. Sci. USA.

[127] Cicchi R., Vogler N., Kapsokalyvas D., Dietzek B., Popp J., Pavone F.S. (2013). From molecular structure to tissue architecture: Collagen organization probed by SHG microscopy. J. Biophotonics.

[128] Wang H., Liang X., Mohammed Y.H., Thomas J.A., Bridle K.R., Thorling C.A., Grice J.E., Xu Z.P., Liu X., Crawford D.H.G. (2015). Real-time histology in liver disease using multiphoton microscopy with fluorescence lifetime imaging. Biomed. Opt. Express.

[129] Wilson D.F. (2017). Oxidative phosphorylation: Regulation and role in cellular and tissue metabolism. J. Physiol..

[130] Chan T.S., Cassim S., Raymond V.A., Gottschalk S., Merlen G., Zwingmann C., Lapierre P., Darby P., Mazer C.D., Bilodeau M. (2018). Upregulation of Krebs cycle and anaerobic glycolysis activity early after onset of liver ischemia. PLoS ONE.

[131] Datta R., Gillette A., Stefely M., Skala M.C. (2021). Recent innovations in fluorescence lifetime imaging microscopy for biology and medicine. J. Biomed. Opt..

[132] Shirshin E.A., Shirmanova M.V., Gayer A.V., Lukina M.M., Nikonova E.E., Yakimov B., Budylin G.S., Dudenkova V.V., Ignatova N.I., Komarov D.V. (2022). Label-free sensing of cells with fluorescence lifetime imaging: The quest for metabolic heterogeneity. Proc. Natl. Acad. Sci. USA.

[133] Verma B.K., Subramaniam P., Vadigepalli R. (2019). Model-based virtual patient analysis of human liver regeneration predicts critical perioperative factors controlling the dynamic mode of response to resection. BMC Syst. Biol..

[134] Verma B.K., Subramaniam P., Vadigepalli R. (2018). Modeling the dynamics of human liver failure post liver resection. Processes.

[135] Verma B.K., Subramaniam P., Vadigepalli R. Characterizing different class of patients based on their liver regeneration capacity post hepatectomy and the prediction of safe future liver for improved recovery. Proceedings of the International Conference on Bioinformatics and Systems Biology.

